# InMut-finder: a software tool for insertion identification in mutagenesis using Nanopore long reads

**DOI:** 10.1186/s12864-021-08206-9

**Published:** 2021-12-19

**Authors:** Rui Song, Ziyao Wang, Hui Wang, Han Zhang, Xuemeng Wang, Hanh Nguyen, David Holding, Bin Yu, Tom Clemente, Shangang Jia, Chi Zhang

**Affiliations:** 1 College of Grassland Science and Technology, China Agricultural University, Beijing, 100193 China; 2 Department of Agronomy and Horticulture, Center for Plant Science Innovation, Beadle Center for Biotechnology, University of Nebraska, Lincoln, NE 68588 USA; 3 Center for Plant Science Innovation, University of Nebraska-Lincoln, Lincoln, NE 68588 USA; 4 School of Biological Sciences, Center for Plant Science Innovation, Beadle Center for Biotechnology, University of Nebraska, Lincoln, NE 68588 USA

## Abstract

**Background:**

Biological mutagens (such as transposon) with sequences inserted, play a crucial role to link observed phenotype and genotype in reverse genetic studies. For this reason, accurate and efficient software tools for identifying insertion sites based on the analysis of sequencing reads are desired.

**Results:**

We developed a bioinformatics tool, a Finder, to identify genome-wide Insertions in Mutagenesis (named as “InMut-Finder”), based on target sequences and flanking sequences from long reads, such as Oxford Nanopore Sequencing. InMut-Finder succeeded in identify > 100 insertion sites in *Medicago truncatula* and soybean mutants based on sequencing reads of whole-genome DNA or enriched insertion-site DNA fragments. Insertion sites discovered by InMut-Finder were validated by PCR experiments.

**Conclusion:**

InMut-Finder is a comprehensive and powerful tool for automated insertion detection from Nanopore long reads. The simplicity, efficiency, and flexibility of InMut-Finder make it a valuable tool for functional genomics and forward and reverse genetics. InMut-Finder was implemented with Perl, R, and Shell scripts, which are independent of the OS. The source code and instructions can be accessed at https://github.com/jsg200830/InMut-Finder.

**Supplementary Information:**

The online version contains supplementary material available at 10.1186/s12864-021-08206-9.

## Background

With the advancement of functional genomics, induced mutagenesis has been widely used in the research of forward and reverse genetics. Mutagenesis techniques include physical mutagens, such as γ-radiation [1], chemical mutagens, such as ethyl methane sulfonate (EMS), and biological mutagens, such as transposable elements (TE) and transfer DNA (T-DNA) [2]. It is feasible and convenient to link T-DNA and transposon-based insertion mutagenesis to observed phenotype in plants in reverse genetics [3–5]. Traditional identification methods for transposon insertion sites are based on polymerase chain reaction (PCR), such as thermal asymmetric interlaced PCR (TAIL-PCR) [6], arbitrarily primed PCR [7], touchdown PCR [8], and vectorette PCR [9]. For example, *Mu* insertion sites on the whole genome in maize were identified in an optimized TAIL-PCR (MuTAIL) which amplified the flanking sequence tags (FSTs) for clone sequencing [10]. Mu-seq, which mapped the *Mu* insertions through three rounds of PCRs based on universal primers, and Illumina sequenced Mu-seq reads are aligned to reference genome using parallel BLASTN in a large population of maize plants [11]. These PCR-based methods required complicated experiment operations and may fail due to the complexity of plant genomes. Moreover, it is difficult to scale up for high throughput, so the whole genome sequencing strategy is very necessary to develop bioinformatic tools for the genome-wide identification of multiple insertion sites of transposable elements. Next-generation sequencing was also used to identify insertions. For example, software tools, such as Transposon Insertion Finder (TIF) [12], RelocaTE2 [13], and panISa [14]. RelocaTE2 is a successful bioinformatics tool based on the Illumina short reads, which filtered out junction reads containing partial TE sequence and flanking genomic sequence, and then searched the flanking sequence against the whole genome of the host organism to determine the insertion sites of TEs. As the Illumina reads (< 250 bp) are too short to cover the complete TE fragment, panISa adopted a structural variant detection strategy to infer the insertion sites, while it selected the clipped reads, i.e., the reads with only a part (similar to flanking sequence) mapped to the reference genome at the potential insertion sites, to determine the potential boundaries of insertion sites based on the two clipped reads in opposite directions. Their sensitivity and precision are limited due to read length and coverage depth, as the short reads need to meet the length requirement of both TEs and flanking sequences to precisely make the conclusion.

Long reads from PacBio and Nanopore are the most suitable way because the average length of reads reaches more than 10 kb, and hence can cover the whole insertion sequence. LoRTE was developed based on PacBio long reads [15], but only tested in the animal of *Drosophila melanogaster*, not in plants. Human-specific LINE-1 insertions were identified by PALMER using PacBio long reads [16], and xTea identified TE insertions (for example Alu, LINE-1, and SVA) from the three different types of sequencing data sets: Illumina pair-end short reads, 10X linked reads, and PacBio/Nanopore long reads [17]. PALMER and xTea depend on the alignment bam files of long reads against the genome reference and were tested in humans only. Both PALMER and xTea can thoroughly identify all different types of LINE-1 insertions or all TE insertions, instead of a specific insertion generated by mutagenesis techniques, which may cause issues for linking to phenotypes. Nanopore sequencing cost is becoming cheaper and cheaper, and is more popular in genome sequencing, compared to PacBio. Previously, we developed an enrichment protocol and computational pipeline to identify *Ds* transposon insertion sites in soybean, by using an oligo probe to capture and sequence DNA fragments containing *Ds* element based on the MinION-based platform of Nanopore [18]. Here, we further developed the computational pipeline to a convenient bioinformatic tool that is efficient to identify genome-wide insertion sites, and we tested its performances in the mutant lines of soybean with enrichment and *Medicago truncatula* without the enrichment step. This software tool is available to the public and can be used for any organism.

## Implementation

### Nanopore sequencing for mutants of *M. truncatula* and soybeans

The mutant plants of *M. truncatula* were grown at the China Agricultural University, and the seeds were originally received from the database of *M. truncatula* mutants (https://medicago-mutant.dasnr.okstate.edu/mutant/index.php). Leaf samples were collected from mutant plants of *M. truncatula*, before being frozen in liquid nitrogen and stored at − 80 °C. DNAs were extracted from the leaf by the QIAGEN®Genomic DNA Extraction Kit (Qiagen, Hilden, Germany). Purified DNA samples were prepared for library construction according to the protocol from genomic sequencing kit SQK-LSK109 (Oxford Nanopore Technologies, Oxford, UK), and sequenced in Nanopore GridION X5 platform. The mutant plants of soybean were from Clement lab at the University of Nebraska - Lincoln, and accessible upon request. The genomic DNA preparation, enrichment by probes, and Nanopore sequencing in soybean mutant lines could be found in our previous paper [18].

### Operating environment

**Fig. 1:**
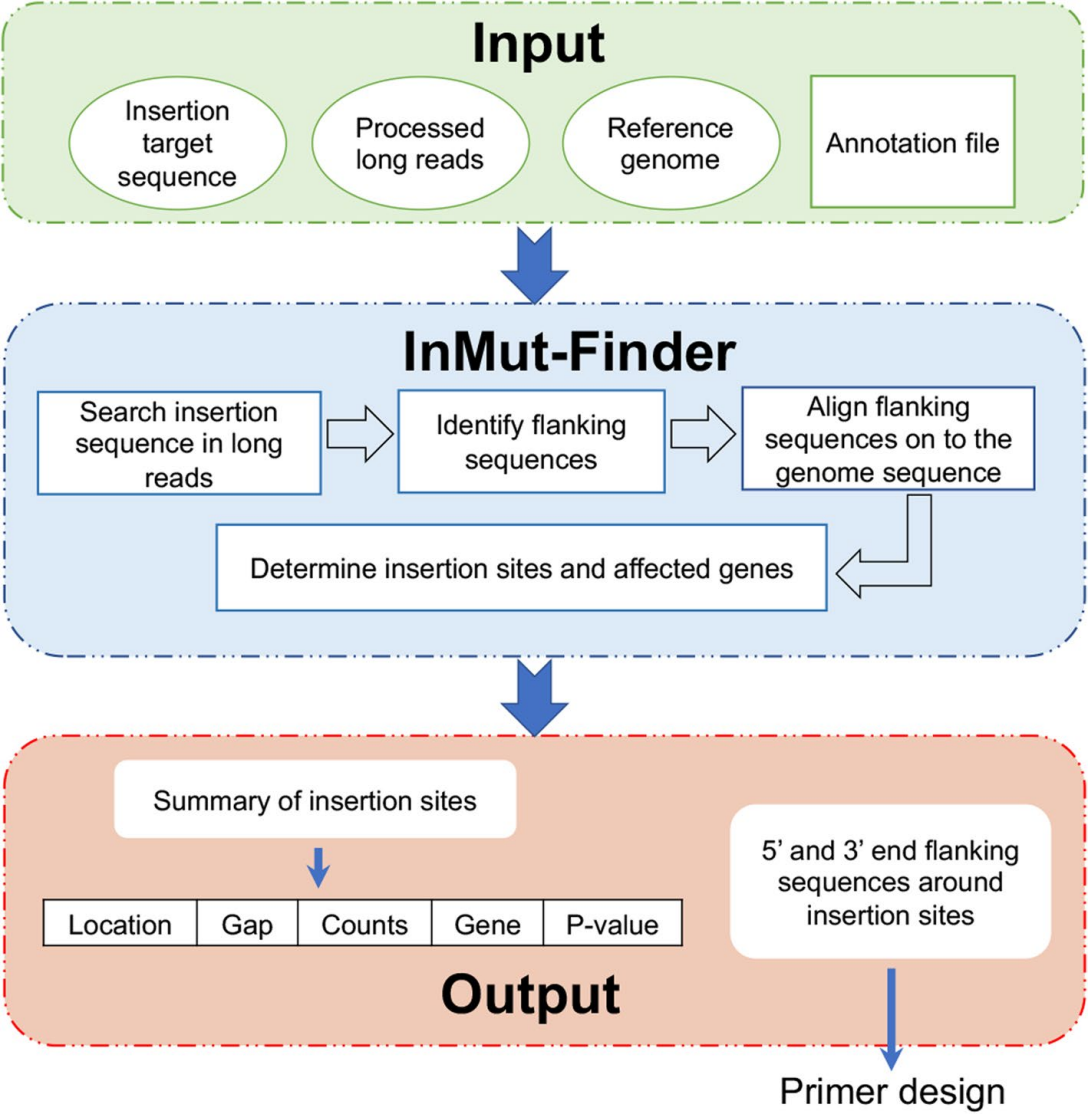
Work flow and verification for InMut-Finder. Work flow for InMut-Finder. Four input files are needed, and two output files are produced. Four scripts work on the four steps in InMut-Finder

**Fig. 2:**
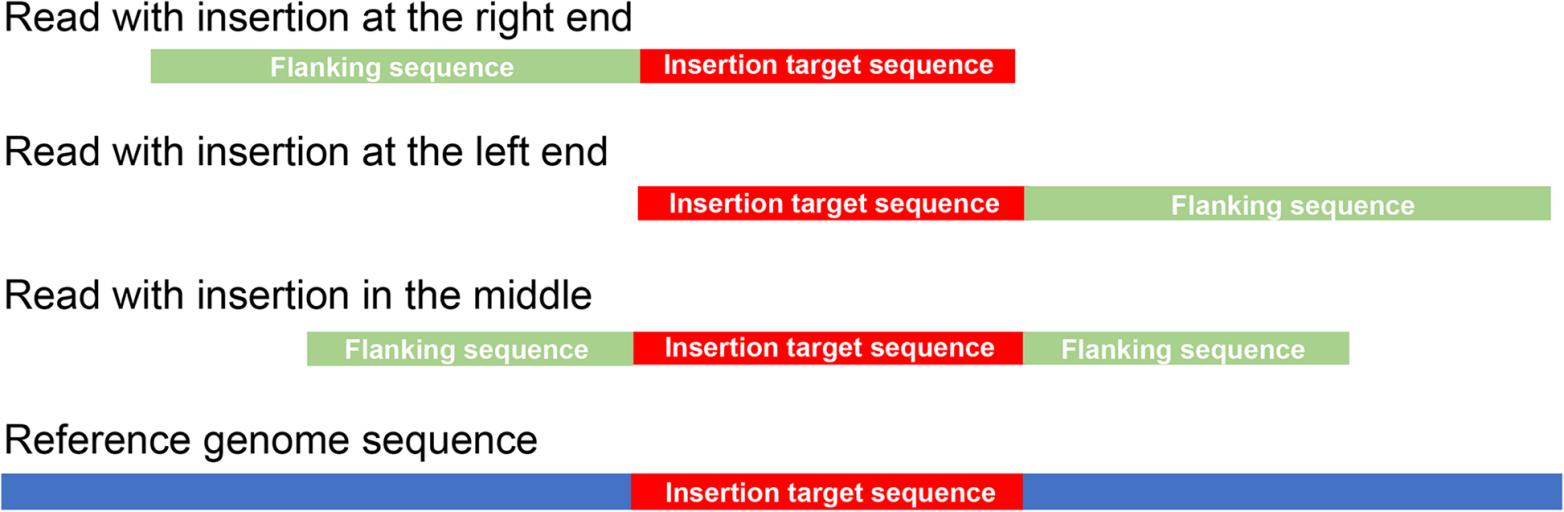
Orientation of flanking sequences neighboring to insertions in long reads

We developed a user-friendly software package, for automatically finding genome-wide Insertions in Mutagenesis (named as “InMut-Finder”) from Nanopore long reads. The main program of InMut-Finder is developed in Perl, R, and Shell scripts. InMut-Finder current version is independent to the operating systems (Linux, Mac OS, and MS Windows). This software tool needs the following dependencies of BLASTN and R, but itself does not need to be installed. There are five major steps (Fig. 1): (i) screen the target sequence against preprocessed long reads with BLASTN using a cutoff of E-value = 10^− 3^; (ii) flanking sequences connected to inserted sequences are identified based on their topology and orientation (Fig. 2); (iii) Align flanking sequences against the reference genome sequence with BLASTN, and uniquely aligned hits with the default cutoff of aligned length > 200 bp and 80% sequence identity are kept; (iv) peaks of aligned flanking sequences, insertion sites and their parent genes in the genome can be determined based on the end of flanking sequences neighboring to insertion sequences; and (v) the zero-inflated Poisson regression is used to model read count data to determine the significance. However, Users could achieve insertion sites of TEs (such as transposon, T-DNA) conveniently in the command line, with only one command by InMut-Finder.

The shell script of “run_command.sh” integrated all the parameters and run all the steps in the command line, after all the input files and parameters are set up in this shell script. It will call “identify_target_in_reads_uniqreadid.pl” to search for the long reads covering both insertion target fragment and flanking sequences, based on BLASTN results. The file of “identify_flanking_in_genome_uniq.pl” works on screening the whole genome for the genomic coordinates of insertion and neighboring genes. The R code of “cal_pscore.r” calculates the *P* values for each insertion, and outputs the final file.

The implementation of InMut-Finder, full documentation and a downloadable test dataset are freely available at https://github.com/jsg200830/InMut-Finder.

## Results and discussion

### Data import and output

This tool requires four input files, including (1) DNA sequence of the inserted target element, for example, *Tnt1* or *Ds* transposons, (2) demultiplexed and trimmed long reads, (3) the sequence of the reference genome, and either FASTA or FASTQ format for query reads and the reference sequences, are allowed in the input of InMut-Finder, (4) genomic annotation file (GFF). The main program is a shell script called ‘run_command.sh’, which would implement all the operations of InMut-Finder in the command line. This is a shell script and needs to be edited to specify input file names and their directories, as well as other parameters. The default parameters have been optimized, but users can modify them to fit their specific tasks. Example data is included in the example_data folder, including all required four files.

InMut-Finder generated two output files. The first one contains the summary of insertion sites, which includes the information of genomic coordinates of insertion, extend of insertion, read counts to support this insertion, neighbor genes within a distance of 2000 bp and *P*-value. The second contains the 5′ and 3′ sequences from the Nanopore sequencing for each insertion. The sequences could be used for designing primers to validate the insertion.

### Enrichment-based insertion identification in soybean

**Fig. 3:**
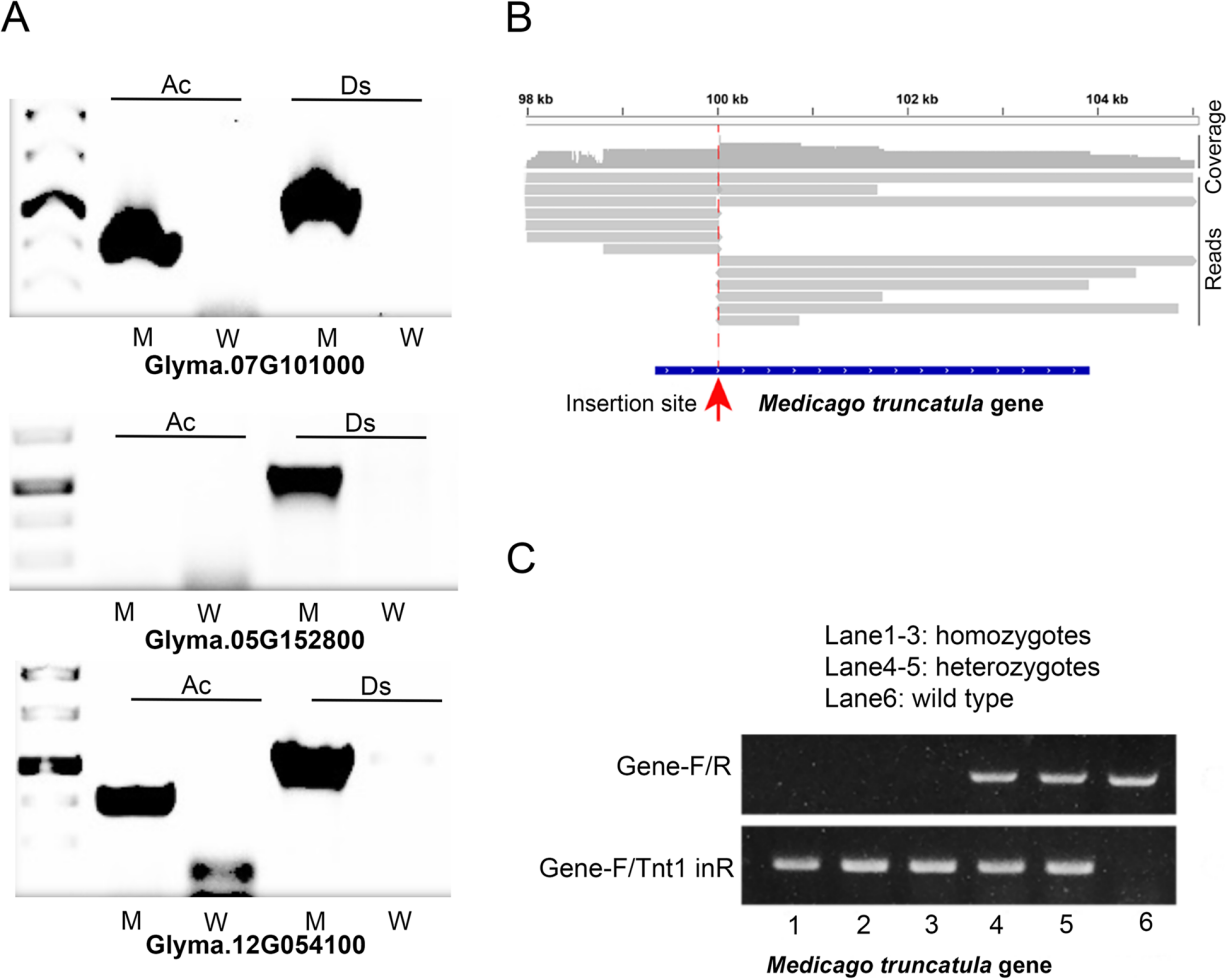
PCR validation of insertions in soybean and *M. truncatula*. **A**, PCR results of three insertions in an Ac-Ds hybrid mutant line of soybean. Ac, primers of Ac-F and Ac-R to test the presence of *Ac* elements; Ds, gene-specific primer + DsL primer to test the presence of *Ds* insertion in the soybean genome. **B**, Nanopore reads are mapped to the reference of *M. truncatula*, and one insertion is shown exactly inside gene 1. **C**, The insertion of *Tnt1* in gene 1 was verified by PCR and agarose gel running in six *M. truncatula* lines, with three alleles of wild type (lane 6), heterozygotes (lane 4 ~ 5), and homozygotes (lane 1 ~ 3)

InMut-Finder can identify insertion locations based on long reads for enriched DNAs in a large genome, such as soybean (~ 979 Mb, Glycine_max_v4.0), by following our previously developed protocol [18]. The enriched DNAs from multiple samples can be pooled with one single barcode and libraries with multiple bar codes can be pooled into one flowcell of MinION sequencer. The protocol was applied to 56 soybean lines in one flowcell, with a design of eight barcodes and seven samples per barcode. InMut-Finder identified 915 to 12,216 high-quality long reads per barcode, which were used to determine 158 to 3096 insertion sites. The maximal read count for an insertion site was 2320. Out of the 56 lines, one soybean mutant line was created by hybridizing Ac-inserted line 1 to Ds-inserted line 2, and Nanopore sequencing was performed with *Ds* probe enrichment (Table 1). For this mutant, there were a total of 1350 insertion sites (Supplementary Table S1), and three insertions were randomly selected for PCR validation. Two pairs (forward + reverse) of primers were used, and they are Ac-F + Ac-R for presence of *Ac* elements and gene-specific primer+DsL for the *Ds* insertion into genes (Supplemental Table S2). The results showed that the positive bands were observed for *Ds* insertion in all the three insertions in the mutant line, but not in the wild type (Fig. 3A), while *Ac* primer pairs only produced bands in two genes of Glyma.07G101000 and Glyma.12G054100, but not in the gene of Glyma.05G152800 and the wild type.

**Table 1:** Summary of reads for enrichment-based sequencing and whole genome sequencing

	Enrichment-based in soybean	Without enrichment in ***M. truncatula***
**Raw reads**	3.9 million	2.4 million
**Average read length (bp)**	1004	7307
**N50 (bp)**	1112	2254
**Tag-inserted reads**	49,271	2891
**Average read length (bp)**	1307	9068
**N50 (bp)**	1423	837
**Flanking sequences**	77,527	3695
**Average length (bp)**	401	3781
**N50 (bp)**	654	98

In order to avoid laborious PCR experiments, we designed a schema based on the combination of multiple barcoding (Supplementary Fig. S1). In this design, 8 samples can be pooled in one barcoding sequencing, and a total of 13 barcodes are needed for 56 samples in one single Nanopore flowcell. In this design, each sample was present in the two barcodes, and sequenced twice. All insertion sites in one specific mutant sample could be identified if these insertions discovered by InMut-Finder appear in two different barcoding groups. For example, the common insertions from two barcodes of BC01 and BC09 should come from the sample “a1”.

### Insertion identification in *Medicago truncatula* without enrichment

Furthermore, InMut-Finder was tested on a whole-genome sequencing dataset to a mutant line of *Medicago truncatula* with Tnt1 inserted. The length of Tnt1 target sequence is 5334 bp, and the genome size of *M. truncatula* is 412.8 Mbp (Version: MedtrA17_4.0). Totally, 2,414,666 Nanopore long reads were produced, with an average length of 7307 bp and an average depth of 44 (Table 1). A total of 2891 long reads were identified with insertion sequence, and the average length of these reads is 9068 bp. Finally, 122 insertion sites were determined (Supplementary Table S3), based on the cutoff of minimal read number ≥ 1, and the maximal abundance of reads of an insertion site was 16. A total of 22 insertion sites occurred in the intergenic regions, and 23 ones were with more than one gene involved. The average length of long reads that were used to identify insertion sites is 9068 bp, and the average length of flanking sequences was 3781 bp, which are longer than 401 bp in the enrichment-based strategy in soybean. In fact, a total of 77,527 flanking sequences in soybean are much more than 3695 ones in *M. truncatula*. Based on these comparison results, we suggested that for most model plants with relatively small genome sizes, whole genome sequencing is preferred for insertion determination by InMut-Finder, while the enrichment strategy is more suitable for large-genome plant species.

Figure 3B shows an example of an insertion site in an *M. truncatula* gene, which has 13 reads for the flanking sequence. PCR experiments were used to validate the selected insertion sites within this gene, for three genotypes of homozygotes, heterozygotes, and wild type (Fig. 3C). Compared to the enrichment strategy in soybean, whole-genome resequencing in *M. truncatula* avoided the ordering of biotin-labeled primers and the laborious enrichment step, and produced longer Nanopore reads with target insertion, although their read number declined.

InMut-Finder employs BLASTN to do the alignment, which allows the running at low memory and multiple threads. Its running could efficiently be finished in one day on one large ONT dataset with > 2 million reads. The insertion site of transposon keeps the randomness and instability at chromosomes in biological mutagens. Therefore, efficient and accurate identification of transposon insertion sites in InMut-Finder is extremely necessary for the screening of functional genes.

## Conclusion

Long-read sequencing technology of Nanopore enables reads to cover the entire insertion sequence, and with the improved sequencing throughput, it allows the genome-wide screen of insertion sites for functional genomics studies. To facilitate research in this emerging field, we developed InMut-finder, a tool for mapping insertion sites of TEs (such as transposon, T-DNA), which is run in the command line. InMut-finder, as a high-throughput and fast tool, is suitable for any species to identify the insertion site of TEs and corresponding neighbor genes, based on the whole genome resequencing or enrichment sequencing with Nanopore technology. This tool may help applications of mutagenesis reach their full potential in life science research.

## Availability and requirements

- Project name: InMut-Finder.
- Project home page: https://github.com/jsg200830/InMut-Finder
- Operating system(s): Linux, Mac, and Windows.
- Programming language: Perl, Shell, and R.
- Other requirements: BLASTN.
- License: GNU General Public License version 2.
- Any restrictions to use by non-academics: None.

## Supplementary Information

**Additional file 1: Supplemental Figure S1**. Study design for multiple barcoding in one single Nanopore flowcell. BC01 ~ BC13 indicates the barcodes in Nanopore sequencing, and a total of 56 samples, a1 ~ a7, b1 ~ b7, c1 ~ c7, d1 ~ d7, e1 ~ e7, f1 ~ f7, g1 ~ g7, and h1 ~ h7, are pooled in 13 barcodes. Each sample presents twice in one flowcell.

**Additional file 2: Supplemental Table S1**. All insertion sites identified in one soybean *Ds* mutant. **Supplemental Table S2**. Three selected insertion sites and primer sequences for PCR validation for the soybean mutant line. **Supplemental Table S3**. All insertion sites identified in one *Tnt1* mutant of *Medicago truncatula*.

## Data Availability

InMut-Finder is freely available at GitHub (https://github.com/jsg200830/InMut-Finder), and the example data is included. The mutant seeds of *Medicago truncatula* were received from the database of *M. truncatula* mutants (https://medicago-mutant.dasnr.okstate.edu/mutant/index.php), and the mutant seeds of soybean were from Clement lab at the University of Nebraska - Lincoln, and accessible upon request.

## Abbreviations

EMS: Ethyl methane sulfonate
TE: Transposable elements
DNA: Deoxyribonucleic acid
T-DNA: Transfer deoxyribonucleic acid
PCR: Polymerase chain reaction
TAIL-PCR: Thermal asymmetric interlaced polymerase chain reaction
*Mu*: Mutator
FSTs: Flanking sequence tags
Mu-seq: Mutant sequencing
BLASTN: Basic local alignment search tool for nucleotide
PacBio: Pacific biosciences
*Ac*: Activator
*Ds*: Dissociation
OS: Operating system
GFF: General feature format
