# Supplementary material for: InMut-finder: a software tool for insertion identification in mutagenesis using Nanopore long reads
Source: BMC Genomics. 2021 Dec 19;22:908. doi: 10.1186/s12864-021-08206-9 (PMC8684674; doi:10.1186/s12864-021-08206-9)
Supplement: Supplementary file 1 — Additional file 1: Supplemental Figure S1. Study design for multiple barcoding in one single Nanopore flowcell. BC01 ~ BC13 indicates the barcodes in Nanopore sequencing, and a total of 56 samples, a1 ~ a7, b1 ~ b7, c1 ~ c7, d1 ~ d7, e1 ~ e7, f1 ~ f7, g1 ~ g7, and h1 ~ h7, are pooled in 13 barcodes. Each sample presents twice in one flowcell. [file 12864_2021_8206_MOESM1_ESM.pdf]

Study design for multiple barcoding in one single Nanopore run

BC01	BC02	BC03	BC04	BC05	BC06	BC07	BC08	BC09	BC10	BC11	BC12	BC13
a1	b1	c1	d1	e1	f1	g1	h1	a1	a2	a3	a4	a5
a2	b2	c2	d2	e2	f2	g2	h2	b1	b2	b3	b4	b5
a3	b3	c3	d3	e3	f3	g3	h3	c1	c2	c3	c4	c5
a4	b4	c4	d4	e4	f4	g4	h4	d1	d2	d3	d4	d5
a5	b5	c5	d5	e5	f5	g5	h5	e1	e2	e3	e4	e5
a6	b6	c6	d6	e6	f6	g6	h6	f1	f2	f3	f4	f5
a7	b7	c7	d7	e7	f7	g7	h7	g1	g2	g3	g4	g5
b7	c7	d7	e7	f7	g7	h7	a7	h1	h2	h3	h4	h5
